# Aggressive Nodular Malignant Melanoma

**DOI:** 10.7759/cureus.16819

**Published:** 2021-08-01

**Authors:** Daniel J Myers, Ethan A Hyde

**Affiliations:** 1 Dermatology, Northwestern University Feinberg School of Medicine, Chicago, USA; 2 Biomedical Sciences, West Virginia School of Osteopathic Medicine, Lewisburg, USA; 3 Department of Podiatry, St. Mary Mercy Medical Center, Livonia, USA

**Keywords:** malignant, melanoma, breslow's depth, nodular, clark level

## Abstract

A subtype of malignant melanomas, nodular melanoma often carries a poor prognosis because of local invasion and frequent distant metastasis. Here, we report a case of progressive dyspnea due to one of the largest primary melanomas in the literature to date along with management strategies and elucidate some of the reasons why patients delay seeking care.

## Introduction

Cutaneous melanoma is a tumor produced by the malignant transformation of melanocytes that derive from the neural crest. Consequently, the disease usually occurs on the skin and occasionally in other areas where neural crest cells are found such as the GI tract or brain [1]. It accounts for 1-3% of all malignancies and 1-2% of all cancer deaths, and its incidence is rapidly increasing worldwide due to improved diagnostics, aging population, and indoor tanning devices [1]. The four major types of melanoma are classified according to the growth pattern, namely, superficial spreading melanoma which constitutes approximately 70% of melanomas; lentigo maligna melanoma which represents 4-10% of melanomas and is often larger than 3 cm, flat, and tan, with marked notching of the borders; acral lentiginous melanoma which constitutes 2-8% of melanomas with lesions that are brown or black and ulcerate in later stages; and finally, nodular melanoma which accounts for approximately 15-30% of melanoma diagnoses; these tumors are typically blue-black but may lack pigment [2]. Nodular melanoma is a significant contributor to the mortality associated with cutaneous melanoma compared to other subtypes. Although accounting for 15% of invasive melanomas, it is responsible for 40% of melanoma-related deaths. This is due to its propensity to present with greater thickness, a faster mitotic growth rate, and earlier metastasis to other vital organs than other subtypes of melanoma, which often results in a poor prognosis [3]. Nodular melanoma most commonly occurs on the trunk in men and on the legs in women [4]. Risk factors include the presence of multiple atypical nevi, family history, fair skin, and excessive sun exposure before the age of 18. The onset is typically during mid-life, and the duration from symptoms to diagnosis ranges from two months to two years [5]. Here, we present a case of progressive dyspnea caused by a large aggressive and invasive nodular melanoma.

## Case presentation

**Figure 1:**
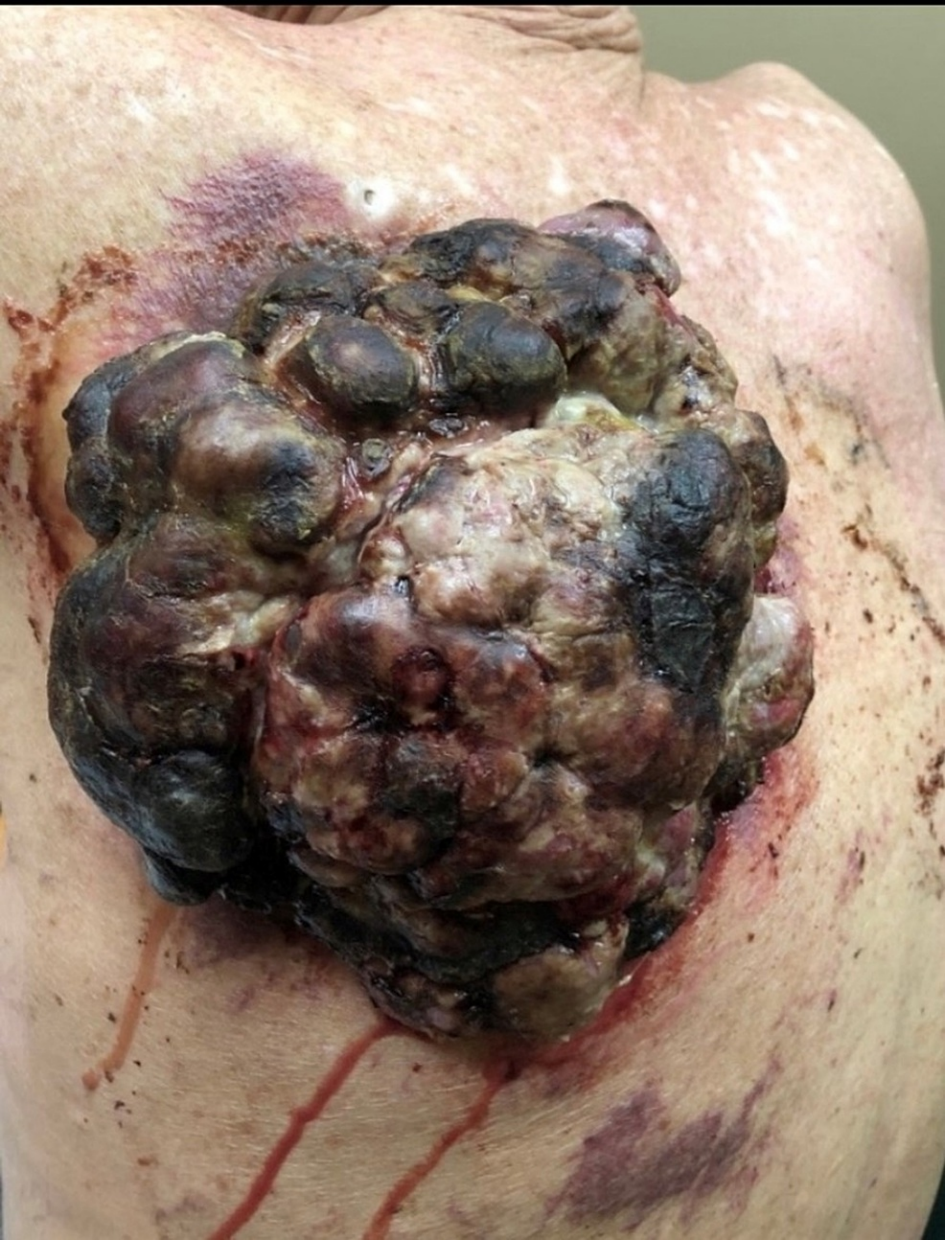
A large 17 × 17 × 6 cm exophytic mass of the left posterior torso consistent with nodular melanoma.

A 69-year-old Caucasian male was admitted to our hospital with complaints of progressively worsening fatigue and dyspnea for two months that were aggravated on exertion. His medical history was significant for chronic emphysema poorly controlled with intermittent inhaler usage, and his social history was notable for an 80-pack-year history of tobacco use, although he discontinued using tobacco products one month ago. The patient had not been to a physician in years. In addition to his dyspnea, the patient noted a large bleeding posterior back mass that began as a small erythematous papule five years ago which had progressively enlarged. On general physical examination, his vitals were within normal limits. He appeared in respiratory distress with increased work of breathing and scattered rhonchi and wheezes throughout. He looked older than his stated age, and prominent anterior kyphosis was noted. His Patient Health Questionnaire-9 score was zero. The dermatological examination showed Fitzpatrick type 2 skin, uneven skin coloring with yellowish predominance, and a posterior left scapular 17.0 × 17.0 × 6.0 cm exophytic, ulcerating, fungating mass with erythema. In addition, there was evidence of infarction and necrosis with fibrinolysis and purulence throughout (Figure 1).

**Figure 2:**
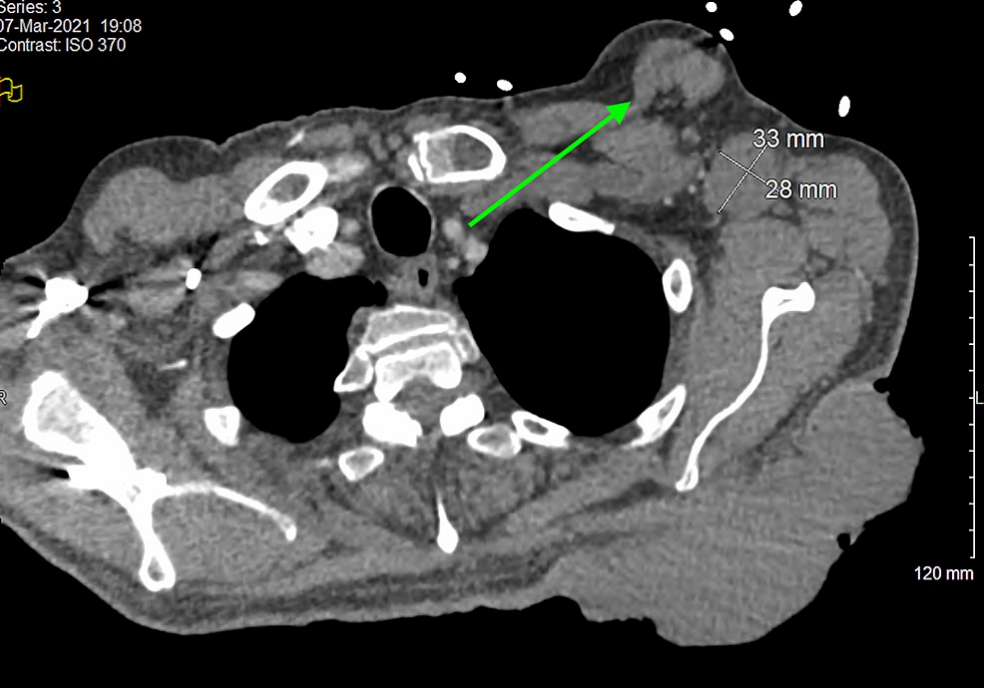
Contrast-enhanced CT image of the thorax. The mass is seen at the level of the scapula with superficial muscle compartment involvement. In addition, moderate pleural effusions and right basilar atelectasis are visualized. CT: computed tomography

**Figure 3:**
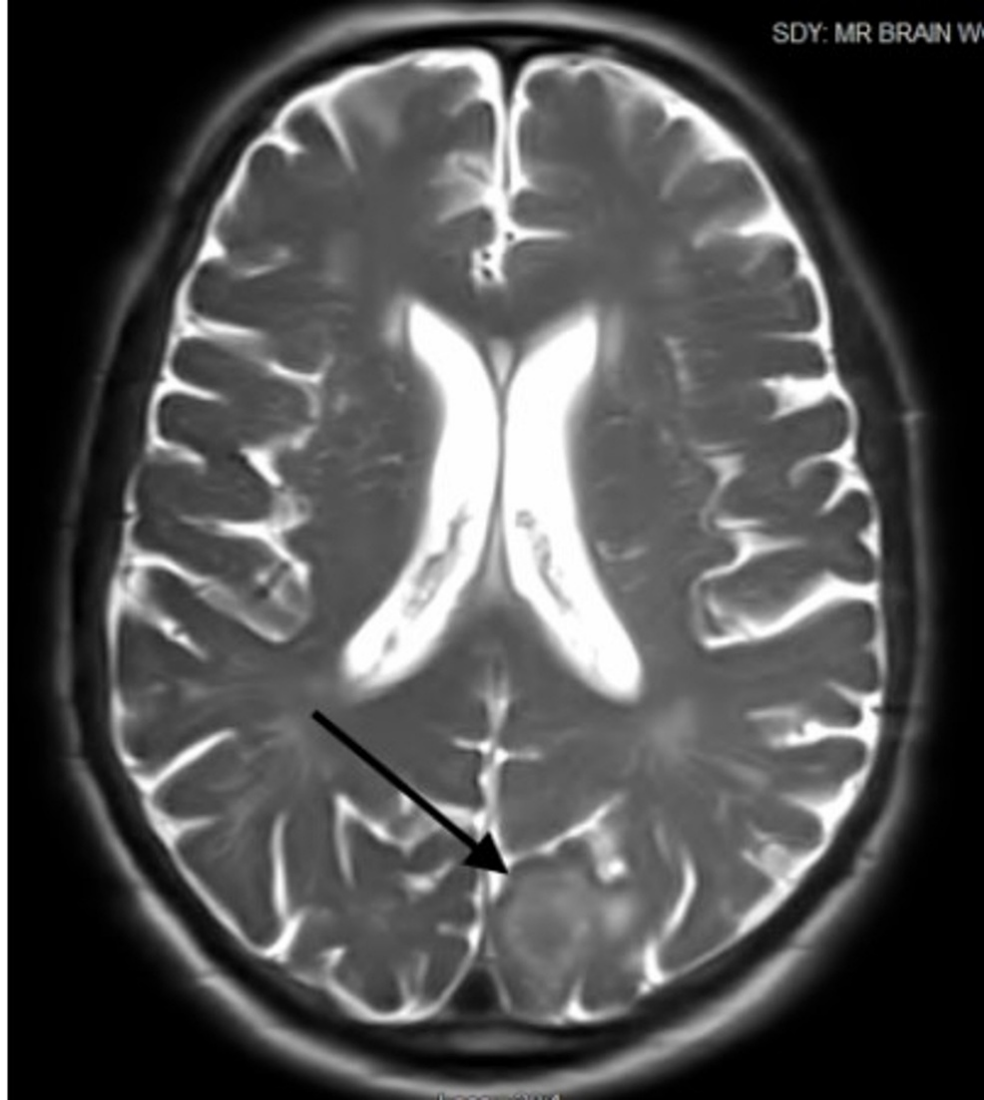
A left occipital lobe-enhancing solid mass measuring 1.5 × 1.7 cm with mild-to-moderate vasogenic edema is seen on axial T2 MRI of the brain. MRI: magnetic resonance imaging

The results of routine laboratory studies of blood and urine were normal except for elevated serum lactate (2.5 mmmol/L), mild normocytic anemia (hemoglobin 9.6 g/dL), and mild thrombocytosis (464,000 platelets/mL) and prothrombin time/international normalized ratio (14.0/2.0). Contrast-enhanced CT of the thorax showed a low-density large subcutaneous mass of the left posterior chest wall with superficial muscle involvement, multiple pulmonary nodules measuring up to 2 cm, moderate emphysema, pleural effusions, and left axillary lymphadenopathy findings, consistent with the presence of extensive metastases (Figure 2). A 1.5-cm round rim-enhancing lesion in the left occipital lobe with a small amount of surrounding vasogenic edema was found on brain MRI, consistent with brain metastasis (Figure 3).

**Figure 4:**
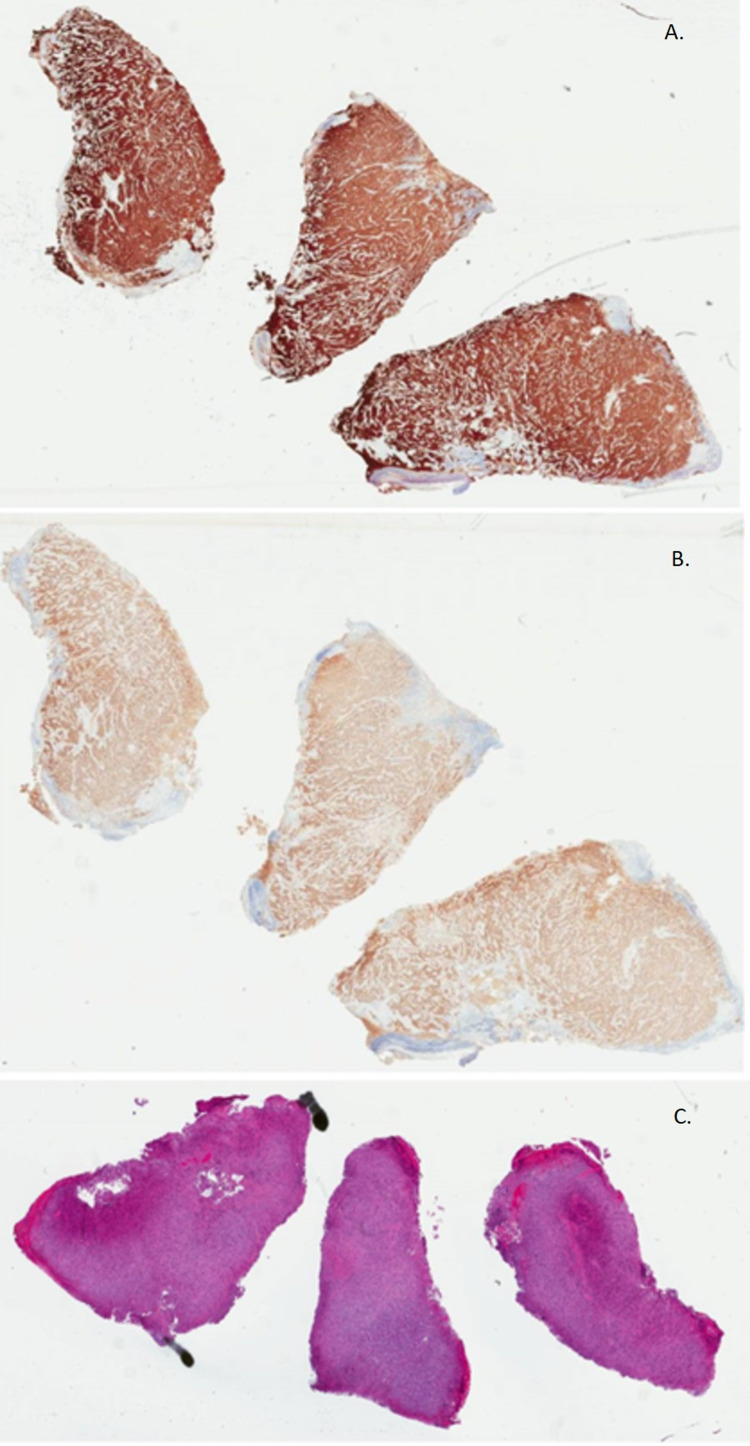
Biopsy of the mass confirming nodular melanoma with a Breslow thickness of 10.1 mm, Clark level IV, stage pT4b, mitotic rate of 3 mitoses/mm². The mass was positive for MelanA (A), SOX-10 (B), and PRAME (C), but negative for BRAFV600.

**Figure 5:**
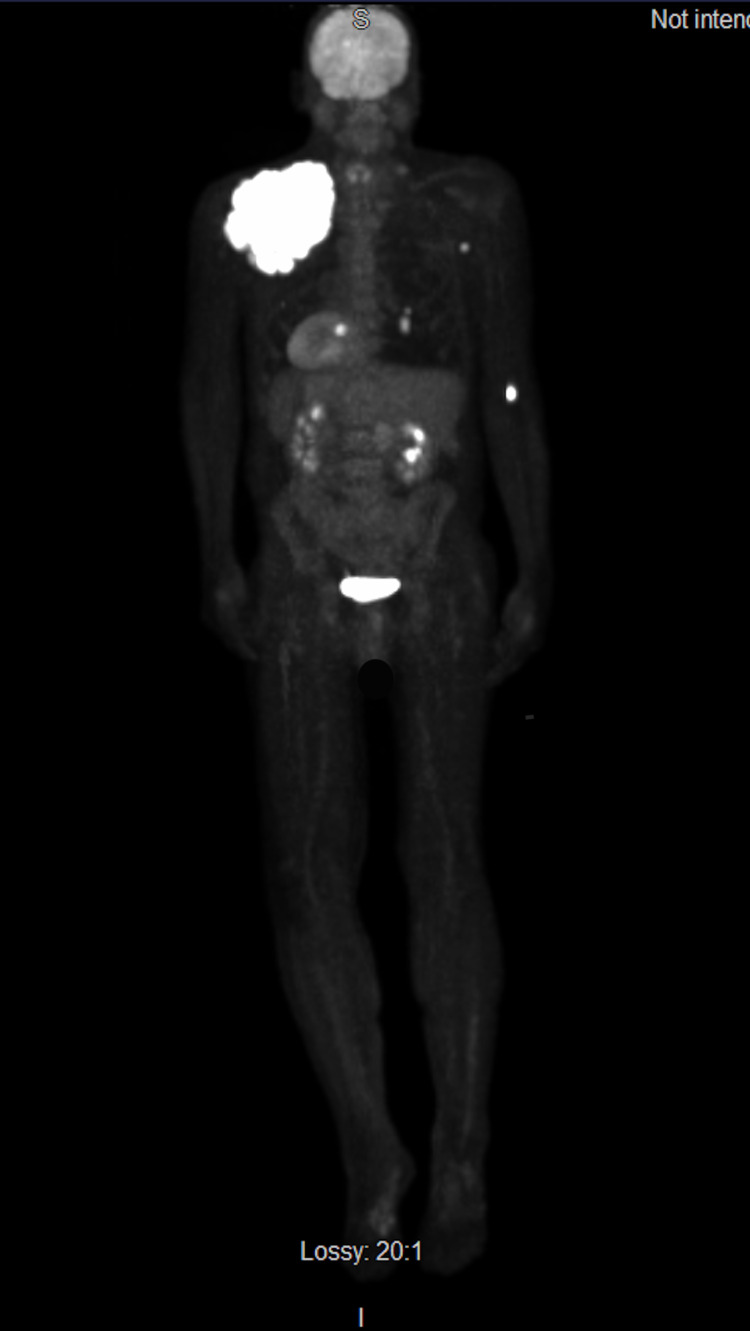
PET-CT displaying disease metastasis to the bilateral axillary lymph nodes, lung, and left plantar bone. PET-CT: positron emission tomography-computed tomography

On biopsy and immunohistochemical stain of the mass, nodular melanoma was confirmed with a Breslow thickness of 10.1 mm, Clark level IV, stage pT4b, mitotic rate of 3 mitoses/mm^2^, and was positive for S100, HMB-45, MelanA, SOX-10, and PRAME, but negative for BRAFV600 with 0% expression of PD-L1 on immunohistochemistry (Figure 4). Positron emission tomography-computed tomography confirmed metastasis to bilateral axillary lymph nodes, lung, and left plantar bone (Figure 5).

After palliative care consultation, the patient elected to pursue all possible treatments. At the time of writing this report, wide resection of the mass was limited by its highly vascular nature. Radiation Oncology initiated radiation therapy using Cyberknife with a cumulative dose of 1,800 cGy directed at the occipital lobe metastasis, and the oncology team evaluated him for enrollment into a clinical trial.

## Discussion

There continues to be an increase in new chemotherapeutics for malignant melanoma [5]. Despite this, therapeutic options remain limited for distant malignant metastatic melanoma [6]. The current mainstay of treatment includes surgical intervention of the primary mass and high-dose interleukin-2, immunotherapy with checkpoint inhibitors directed against cytotoxic T-lymphocyte-associated antigen 4 and programmed cell death protein 1, and inhibition of the mitogen-activated protein kinase pathway with BRAF and mitogen-activated protein kinase inhibitors in patients whose tumors contain a *V600* mutation in the *BRAF* gene [6]. The prognosis is worse in patients with negative BRAFV600 and PD-L1 expression, as seen in our patient. While there are no specific guidelines for distant malignant melanoma, treatment with T-VEC, ipilimumab, and nivolumab; palliative care; or enrollment in a clinical trial are all therapeutic considerations [6]. In this case, the treating team believed clinical trial enrollment was an appropriate option considering his substantial disease burden.

Giant primary cutaneous melanomas have been rarely reported as increased access to primary healthcare and screening measures continue to improve and reduce their incidence [7-11]. After extensive literature review, cases of giant primary melanoma, defined as lesions at least 10 cm in diameter or 48 mm in thickness, are almost exclusively associated with extensive metastatic disease [12]. However, there are some reports of giant nodular melanoma without metastatic disease which may represent a genetically distinct subtype of giant melanoma [12]. In this case, the gross size of the mass represents one of the largest primary cutaneous melanomas with unfortunate widespread metastases reported to date.

Early diagnosis of melanomas is closely related to improved outcomes across all health-related metrics. The reasons for delayed diagnosis are multifactorial and include a lack of knowledge of the disease among the population, as well as a failure of patients and physicians to conduct annual skin examinations. Prior research has shown that 50% of melanomas are found by the patients [13]. Despite this, many patients who present with invasive disease delay seeking care. Cassileth et al. found that patients who experienced changes in size, color, or elevation in a pigmented lesion waited an average of one year before presenting to a physician for evaluation [13]. Bleeding or ulceration resulted in a shorter delay in presentation, but these symptoms were associated with higher Breslow depths on presentation and a poorer prognosis [13]. Our case included both a long duration of illness and significant bleeding before presentation which correlated with the patient’s extensive disease at the time of diagnosis. While the patient refrained from giving a decisive answer on why he did not seek intervention earlier, given the negative depression screen and not having visited a general practitioner in many years, it was thought that he may have delayed pursuing care due to unintentional self-neglect and fear of visiting a doctor once the mass reached a certain size. Interestingly, the primary reason for our patient’s presentation was his progressive dyspnea which likely stemmed from multifactorial etiologies, including mass compression, anemia due to chronic disease and blood loss from the mass, and poorly controlled chronic emphysema.

## Conclusions

Giant malignant melanomas are extremely rare entities and pose a surgical and medical challenge due to their destructive nature and poor prognostication. Even though they have significant metastatic potential, a subset of these tumors could have a more benign course. Additionally, this case highlights the need for developing more therapeutic options in stage four metastatic melanoma and the current management options of primary giant melanomas with distant metastasis. It re-emphasizes the continued need for public health campaigns to recommend annual skin screens by both primary care and dermatologists, dispersing knowledge on how to reduce their risk factors, incentivizing patients to seek diagnosis and treatment as soon as a skin lesion begins to appear atypical, and the possible devastating consequences if not adequately followed promptly.
